# Severe Human Parainfluenza Virus Community- and Healthcare-Acquired Pneumonia in Adults at Tertiary Hospital, Seoul, South Korea, 2010–2019

**DOI:** 10.3201/eid3006.230670

**Published:** 2024-06

**Authors:** Joung Ha Park, Sang-Bum Hong, Jin Won Huh, Jiwon Jung, Min Jae Kim, Yong Pil Chong, Heungsup Sung, Kyung Hyun Do, Sung-Han Kim, Sang-Oh Lee, Yang Soo Kim, Chae-Man Lim, Younsuck Koh, Sang-Ho Choi

**Affiliations:** Chung-Ang University Gwangmyeong Hospital, Gwangmyeong, South Korea (J.H. Park);; University of Ulsan College of Medicine Asan Medical Center, Seoul, South Korea (S.-B. Hong, J.W. Huh, J. Jung, M.J. Kim, Y.P. Chong, H. Sung, K.H. Do, S.-H. Kim, S-.O. Lee, Y.S. Kim, C.-M. Lim, Y. Koh, S.-H. Choi)

**Keywords:** pneumonia, human parainfluenza virus, severe pneumonia, immunocompromised, influenza, respiratory infections, viruses, South Korea

## Abstract

The characteristics of severe human parainfluenza virus (HPIV)–associated pneumonia in adults have not been well evaluated. We investigated epidemiologic and clinical characteristics of 143 patients with severe HPIV-associated pneumonia during 2010–2019. HPIV was the most common cause (25.2%) of severe virus-associated hospital-acquired pneumonia and the third most common cause (15.7%) of severe virus-associated community-acquired pneumonia. Hematologic malignancy (35.0%), diabetes mellitus (23.8%), and structural lung disease (21.0%) were common underlying conditions. Co-infections occurred in 54.5% of patients admitted to an intensive care unit. The 90-day mortality rate for HPIV-associated pneumonia was comparable to that for severe influenza virus–associated pneumonia (55.2% vs. 48.4%; p = 0.22). Ribavirin treatment was not associated with lower mortality rates. Fungal co-infections were associated with 82.4% of deaths. Clinicians should consider the possibility of pathogenic co-infections in patients with HPIV-associated pneumonia. Contact precautions and environmental cleaning are crucial to prevent HPIV transmission in hospital settings.

Human parainfluenza virus (HPIV) is a major cause of acute respiratory tract infections, such as croup in children and pneumonia in immunocompromised patients (*1*). HPIV is a single-stranded, enveloped RNA virus in the Paramyxoviridae family (*2*) and is classified into 4 major serotypes, HPIV-1–4, according to complement fixation and hemagglutinating antigens (*3*). HPIV-1 and HPIV-3 belong to the genus *Respirovirus*, whereas HPIV-2 and HPIV-4 belong to the genus *Rubulavirus*. HPIV-3 is the most common serotype associated with pneumonia and bronchiolitis, and HPIV-1 and HPIV-2 are usually associated with croup in children (*2*).

HPIV infections are generally mild and self-limiting in adults (*4*); however, HPIV-associated pneumonia has been increasingly reported (*5*). HPIV accounts for 2.0%–7.6% of pathogens causing community-acquired pneumonia (CAP) (*6*–*8*) and 3.4%–6.1% of pathogens causing hospital-acquired pneumonia (HAP) (*9*,*10*). Data on the epidemiologic and clinical characteristics of severe HPIV-associated pneumonia in adults are limited. Although a retrospective study described the clinical characteristics of hospitalized adults with HPIV infections, the primary analysis did not focus on patients requiring admission into the intensive care unit (ICU) (*1*). We investigated the epidemiologic and clinical characteristics and outcomes of a large cohort of critically ill adults in South Korea who had severe HPIV-associated pneumonia requiring ICU care during a 10-year period.

## Methods

### Ethics

This study was approved by the Institutional Review Board of Asan Medical Center, Seoul, South Korea (approval no. 2010–0079). Informed consent was waived because of the observational nature of the study.

### Study Design

The study was performed as a part of a prospective, observational cohort study of patients with severe virus-associated pneumonia housed within the 28-bed ICU at Asan Medical Center, a 2,700-bed hospital in Seoul (*6*,*9*,*11*). All 3,099 adult patients included in the study were >16 years of age and admitted to the ICU with severe pneumonia during January 2010–December 2019; they were prospectively enrolled in the study cohort and monitored until discharge or death. We analyzed clinical data from the patients’ electronic medical records, including demographic characteristics, underlying diseases or conditions, clinical manifestations, co-infecting pathogens, and outcomes. To describe clinical outcomes, we compared HPIV-associated pneumonia outcomes with those of severe influenza virus–associated pneumonia. We evaluated outcomes according to the length of hospital stay, duration within the ICU, complications from ventilator-associated pneumonia, and number of deaths.

We performed microbiologic evaluations as previously described (*6*). We identified respiratory viruses from nasopharyngeal swab samples, nasopharyngeal aspirates, bronchial washings, and bronchoalveolar lavage (BAL) fluids by using multiplex reverse transcription PCR (RT-PCR); we used the Anyplex II RV16 Detection Kit during 2010–2017, and the Allplex Respiratory Panel during 2018–2019 (both Seegene Inc., https://www.seegene.com). We tested samples for influenza virus A and B, respiratory syncytial virus A and B, adenovirus, human metapneumovirus, HPIV serotypes 1–4, enterovirus, rhinovirus, and human coronaviruses 229E/NL63, OC43, and HKU1.

### Definitions

We defined pneumonia as a case that had new pulmonary infiltrations observed on a chest radiograph and >2 of the following symptoms: fever, cough, productive sputum, dyspnea, or prescription of antimicrobial drugs for pneumonia by the attending physician. We defined severe pneumonia as a case requiring invasive mechanical ventilation because of respiratory failure or septic shock requiring vasopressors (*12*). We defined HAP as a case occurring >48 hours after hospital admission (*13*) and HPIV-associated HAP as a case for which a respiratory virus RT-PCR was performed >48 hours after hospital admission and positive HPIV results were obtained. We defined the remaining cases as CAP. We designated immunocompromised patients as those who had undergone solid organ transplants, bone marrow transplants, or cytotoxic chemotherapy within 6 months or those who had received immunosuppressants, including corticosteroids, within 1 month before admission to the ICU. We also defined co-pathogens as pathogenic organisms identified within 3 working days of a severe HPIV-associated pneumonia diagnosis.

### Statistical Analyses

We compared categorical variables by using the χ^2^ or Fisher exact test and continuous variables by using the Student *t-*test or Mann-Whitney U test, as appropriate. We expressed continuous data as median values and interquartile ranges. We investigated risk factors for 90-day mortality by using univariate and multivariate logistic regression models. We based the final multivariate model on the univariate analysis and clinical relevance of potential risk factors. We included variables with a p value of <0.20 in the final multivariate model. All significance tests were 2-tailed, and we considered p*<*0.05 statistically significant. We performed all analyses by using SPSS Statistics 23 (IBM, https://www.ibm.com).

## Results

### Epidemiology of Severe HPIV-Associated Pneumonia

**Figure 1:**
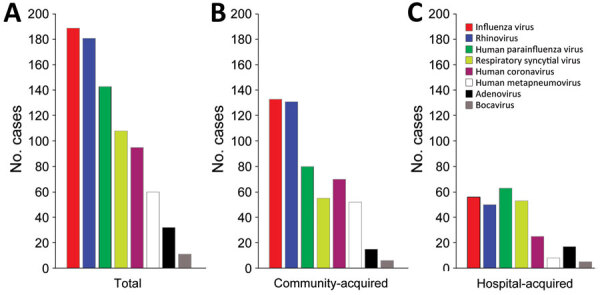
Prevalence of respiratory viruses in study of severe human parainfluenza virus community- and healthcare-acquired pneumonia in adults at a tertiary hospital in Seoul, South Korea, 2010–2019. Number of cases of different respiratory virus infections are given for 760 patients with severe pneumonia admitted to the intensive care unit at Asan Medical Center. A) Total number of patients with indicated virus infections. B) Number of patients with community-acquired virus infections. C) Number of patients with hospital-acquired virus infections.

A total of 2,479 patients with severe pneumonia underwent multiplex RT-PCR testing of their respiratory samples to detect virus infections; pathogenic viruses were identified in 760 patients. Influenza virus was the most common pathogenic virus identified in patients with severe pneumonia; influenza virus was found in 189 (7.6%) patients, followed by rhinovirus in 181 (7.3%) and HPIV in 143 (5.8%) patients. The co-infection rates were 48.7% for influenza virus, 56.9% for rhinovirus, and 54.5% for HPIV among patients with severe virus-associated pneumonia. For severe virus-associated CAP, HPIV was the third most common virus (15.7%), after influenza virus (26.1%) and rhinovirus (25.7%). In contrast, for severe virus-associated HAP, HPIV was the most common virus at 25.2% (Figure 1). 

**Figure 2:**
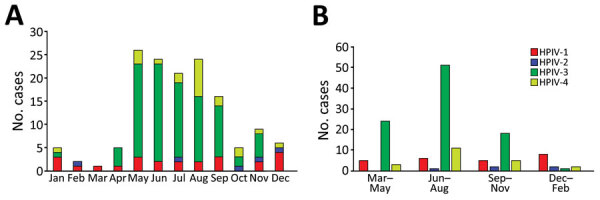
Seasonality of severe HPIV community- and healthcare-acquired pneumonia in adults at a tertiary hospital in Seoul, South Korea, 2010–2019. Colors indicate total number of infection cases caused by 4 parainfluenza virus serotypes during a 10-year period. A) Monthly distribution of severe HPIV-associated pneumonia. B) Seasonal distribution of severe HPIV-associated pneumonia. HPIV, human parainfluenza virus.

Among the 143 patients with severe HPIV-associated pneumonia, 42 underwent bronchoscopic BAL for etiologic diagnosis. Of those patients, 35 had positive HPIV RT-PCR results from BAL samples, whereas 24 showed positive results from both BAL and nasopharyngeal swab samples. Among the 143 patients, HPIV-3 was the most common serotype (65.7%), followed by HPIV-1 (16.1%), HPIV-4 (14.7%), and HPIV-2 (3.5%). The prevalence of HPIV-1, HPIV-2, and HPIV-4 was higher in the CAP group than in the HAP group. HPIV-3 was the dominant serotype during all seasons except winter (December–February); HPIV-1 was the dominant serotype during winter (Figure 2).

### Demographics and Clinical Characteristics

We analyzed demographic and clinical characteristics of the 143 patients with severe HPIV-associated pneumonia (Table 1). The median age of those patients was 61.6 years; 97.2% had underlying illnesses or conditions. CAP was observed in 80 (55.9%) and HAP in 63 (44.1%) patients. Among the 63 HAP patients, the median hospital stay until HAP diagnosis was 22.0 (interquartile range 13.0–40.0) days. Patients with CAP were older than patients with HAP (median 66.0 vs. 55.9 years; p<0.001). 

**Table 1:** Patient characteristics in study of severe HPIV community- and healthcare-acquired pneumonia in adults at a tertiary hospital in Seoul, South Korea, 2010–2019*

Characteristics	All HPIV infections	Community-acquired HPIV	Hospital-acquired HPIV	p value
No. patients	143	80	63	
Sex				0.27
M	89 (62.2)	53 (66.3)	36 (57.1)	
F	54 (37.8)	27 (33.7)	27 (42.9)	
Mean age, y (+SD)	61.6 (+14.0)	66.0 (+11.6)	55.9 (+14.9)	<0.001
HPIV serotype†				0.25
HPIV-3	94 (65.7)	41 (51.2)	53 (84.1)	
HPIV-1	23 (16.1)	18 (22.5)	5 (7.9)	
HPIV-4	21 (14.7)	16 (20.0)	5 (7.9)	
HPIV-2	5 (3.5)	5 (6.3)	0 (0)	
Underlying disease/condition	139 (97.2)	NA	NA	NA
Structural lung disease	30 (21.0)	23 (28.7)	7 (11.1)	0.01
Chronic obstructive lung disease	14 (9.8)	13 (16.3)	1 (1.6)	0.003
Interstitial lung disease	8 (5.6)	6 (7.5)	2 (3.2)	0.47
Bronchiectasis	7 (4.9)	5 (6.3)	2 (3.2)	0.47
Tuberculosis-destroyed lung	1 (0.7)	0 (0)	1 (1.6)	0.44
Hematologic malignancy	50 (35.0)	15 (18.8)	35 (55.6)	<0.001
Diabetes mellitus	34 (23.8)	22 (27.5)	12 (19.0)	0.24
Solid cancer	22 (15.4)	18 (22.5)	4 (6.3)	0.008
End-stage renal disease	7 (4.9)	6 (7.5)	1 (1.6)	0.10
Chronic renal failure, no hemodialysis	6 (4.2)	3 (3.8)	3 (4.8)	>0.99
Liver cirrhosis	4 (2.8)	2 (2.5)	2 (3.2)	>0.99
Congestive heart failure	4 (2.8)	2 (2.5)	2 (3.2)	>0.99
Cerebrovascular attack	6 (4.2)	4 (5.0)	2 (3.2)	0.69
Solid organ transplant‡	6 (4.2)	4 (5.0)	2 (3.2)	0.69
Hematopoietic stem cell transplant	14 (9.8)	6 (7.5)	8 (12.7)	0.30
Immunocompromised status§	88 (61.5)	40 (50.0)	48 (76.2)	0.001
Receipt of immunosuppressant	42 (29.4)	15 (18.8)	27 (42.9)	0.002
Recent chemotherapy	56 (39.2)	22 (27.5)	34 (54.0)	0.001
Active smoker	11 (7.7)	9 (11.3)	2 (3.2)	0.07
Surgery within 1 mo of ICU admission	11 (7.7)	3 (3.8)	8 (12.7)	0.046
Neutropenia¶	39 (27.3)	14 (17.5)	25 (39.7)	0.003
Clinical manifestations				
Mean APACHE II score (+SD)	25.4 (+7.0)	24.6 (+7.3)	26.4 (+6.5)	0.13
Mean SOFA score (+SD)	9.9 (+4.0)	9.15 (+4.1)	10.8 (+ 3.7)	0.01
Septic shock at admission	75 (52.4)	43 (53.8)	32 (50.8)	0.73
Mechanical ventilation	138 (96.5)	75 (93.8)	63 (100.0)	0.07
Treatment				
Oral ribavirin#	67 (46.9)	31 (38.8)	36 (57.1)	0.03
IVIG	36 (25.2)	15 (18.8)	21 (33.3)	0.046

*Values are no. (%) except as indicated. APACHE II, acute physiology and chronic health evaluation II; HPIV, human parainfluenza virus; IQR, interquartile range; IVIG, intravenous immunoglobulin; NA, not applicable; SOFA, sequential organ failure assessment. †Serotypes are listed according to prevalence. ‡Patients with lung (n = 1) or kidney (n = 5) transplants. §Defined as either daily receipt of immunosuppressants (including corticosteroids), human immunodeficiency virus infection, solid organ or hematopoietic stem cell transplant recipients, receipt of chemotherapy for underlying malignancy during the previous 6 months, or underlying immune deficiency disorder. ¶Absolute neutrophil count of <500/mm^3^. #Use of oral ribavirin for >2 days.

Hematologic malignancy (35.0%), diabetes mellitus (23.8%), and structural lung disease (21.0%) were the most common underlying illnesses in the total study population. Chronic obstructive pulmonary disease was more common in the CAP group than in the HAP group (16.3% vs. 1.6%; p = 0.003); hematologic malignancies were more common in the HAP group (18.8% vs. 55.6%; p<0.001). Also, immunocompromised conditions were more common in the HAP group (50.0% vs. 76.2%; p = 0.001). The initial sequential organ failure assessment scores were lower in the CAP group than in the HAP group (9.2 vs. 10.8; p = 0.01). Septic shock was observed in 53.8% of patients with CAP and 50.8% of patients with HAP. Oral ribavirin (38.8% vs. 57.1%; p = 0.03) or intravenous immunoglobulin (IVIG) (18.8% vs. 33.3%, p = 0.046) were prescribed less frequently to patients in the CAP group than in the HAP group.

### Co-Infection

At the time of ICU admission, 78 (54.5%) of the 143 patients with severe HPIV-associated pneumonia were co-infected with other pathogens; 48 (33.6%) patients were co-infected with bacteria, 21 (14.7%) with viruses, and 17 (11.9%) with fungi (Table 2). Among bacterial pathogens, *Pseudomonas aeruginosa* (9/48) was the most common, followed by *Staphylococcus aureus* (8/48), *Klebsiella pneumoniae* (8/48), and *Acinetobacter baumannii* (8/48). Rhinovirus (6/21) and respiratory syncytial virus (5/21) were the most common co-infecting pathogenic viruses. The rates of bacterial (40.0% vs. 25.4%; p = 0.07) and viral (15.9% vs. 14.3%; p = 0.90) co-infections were not different between the CAP and HAP groups. However, fungal co-infections, mainly *Aspergillus* spp., were less common in the CAP group than in the HAP group (5.0% vs. 20.6%; p = 0.004). *Aspergillus* spp. co-infections were found in 13 of 15 patients who had immunocompromised conditions (7 patients had hematologic malignancy, 3 had hematopoietic stem cell transplants, 2 were taking immunosuppressant medications, and 1 had a solid cancer).

**Table 2:** Co-infections of patients in study of severe human parainfluenza virus community- and healthcare-acquired pneumonia in adults at a tertiary hospital in Seoul, South Korea, 2010–2019*

Co-pathogens†	Total, n = 143	Community acquired, n = 80	Hospital acquired, n = 63	p value
Any	78 (54.5)	44 (55.0)	34 (54.0)	0.90
Bacteria	48 (33.6)	32 (40.0)	16 (25.4)	0.07
Gram positive				
* Staphylococcus aureus*	8	4	4	0.73
* Streptococcus pneumoniae*	7	6	1	0.13
* Streptococcus pyogenes*	0	0	0	NA
* Streptococcus agalactiae*	0	0	0	NA
* Corynebacterium striatum*	1	0	1	0.44
Gram negative				
* Haemophilus influenzae*	1	1	0	>0.99
* Moraxella catarrhalis*	0	0	0	NA
*Legionella* sp.	1	1	0	>0.99
Enteric gram-negative bacilli				
* Klebsiella pneumoniae*	8	7	1	0.08
* Escherichia coli*	2	2	0	0.50
* Enterobacter cloacae*	2	2	0	0.50
* Serratia marcescens*	1	1	0	>0.99
* Enterobacter aerogenes*	1	1	0	>0.99
Nonfermenting gram-negative bacilli				
* Pseudomonas aeruginosa*	9	5	4	0.98
* Acinetobacter baumannii*	8	3	5	0.30
* Stenotrophomonas maltophilia*	1	0	1	0.44
Viruses	21 (14.7)	12 (15.0)	9 (14.3)	0.90
Rhinovirus	6	5	1	0.23
Respiratory syncytial virus	5	2	3	0.65
Adenovirus	3	2	1	>0.99
Human coronavirus	2	2	0	0.50
Human metapneumovirus	2	0	2	0.19
Bocavirus	2	0	2	0.19
Influenza virus	1	1	0	>0.99
Fungi	17 (11.9)	4 (5.0)	13 (20.6)	0.004
*Aspergillus* species	15	3	12	0.003
* Pneumocystis jirovecii*	2	1	1	>0.99
Nontuberculous mycobacteria	1 (0.7)	0	1 (1.6)	0.44

*Values are no. (%) except as indicated. NA, not applicable. †Includes pathogens identified from bronchoalveolar lavage fluid or other specimens, such as nasopharyngeal, sputum, endotracheal aspirate, and blood culture samples during admission to the intensive care unit. Categories of co-infection were not mutually exclusive. Some cases were associated with >2 categories of pathogens.

### Outcomes and Risk Factors for 90-Day Mortality Rate

The overall 90-day mortality rate was 55.2% for HPIV-associated pneumonia, which was comparable to 48.4% for severe influenza virus–associated pneumonia (p = 0.22) within the same cohort (Table 3). Ventilator-associated pneumonia occurred in 10.5% (15/143) of the study population (Table 3). The mortality rate after oral ribavirin or IVIG treatment was not lower than that for untreated patients (56.7% vs. 53.9%; p = 0.74). Furthermore, mortality rates after oral ribavirin or IVIG treatment were not lower than those for untreated patients in the immunocompromised (65.6% vs. 62.5%; p = 0.77) or nonimmunocompromised (43.6% vs. 37.5%; p = 0.69) patient groups. Patients with a fungal co-infection had a significantly higher 90-day mortality rate than those with no fungal co-infection (82.4% vs. 51.6%; p = 0.02). In multivariate analysis of risk factors, the adjusted odds ratios (aORs) were 2.21 (95% CI 1.00–4.86) for HAP, 1.29 (95% CI 1.15–1.44) for the initial SOFA score, and 4.64 (95% CI 1.05–20.63) for fungal co-infection, which was significantly associated with 90-day mortality rate (p = 0.04) (Table 4). 

**Table 3:** Patient outcomes in study of severe HPIV community- and healthcare-acquired pneumonia in adults at a tertiary hospital in Seoul, South Korea, 2010–2019*

Patient outcomes	HPIV infections				Influenza virus infections			p value†
	Total, n = 143	CAP, n = 80	HAP, n = 63		Total, n = 189	CAP, n = 133	HAP, n = 56	
Mortality rates								
7 d	20 (14.0)	8 (10.0)	12 (19.0)		16/185 (8.6)	11/132 (8.3)	5/53 (9.4)	0.13
30 d	57 (39.9)	22 (27.5)	35 (55.6)		61/185 (33.0)	41/132 (31.1)	20/53 (37.7)	0.21
60 d	73 (51.0)	30 (37.5)	43 (68.3)		81/185 (43.8)	50/132 (37.9)	31/53 (58.5)	0.21
90 d	79 (55.2)	34 (42.5)	45 (71.4)		89/185 (48.1)	53/132 (40.2)	36/53 (67.9)	0.22
In-hospital	73 (51.0)	29 (36.3)	44 (69.8)		78/185 (42.2)	46/132 (34.8)	32/53 (60.4)	0.10
Mean ICU stay, d (+SD)	17.1 (+19.4)	17.4 (+23.0)	16.7 (+13.7)		17.6 (+17.3)	NA	NA	0.82
Mean hospital stay, d (+SD) ‡	50.1 (+47.4)	39.5 (+40.5)	63.6 (+52.2)		53.1 (+71.4)	NA	NA	0.66
Ventilator-associated pneumonia	15 (10.5)	4 (5.0)	11 (17.5)		25 (13.3)	15 (11.3)	10 (17.9)	0.44

*Values are no. (%) except as indicated. Denominators are different in some cases because of missing data. CAP, community-acquired pneumonia; HAP, hospital-acquired pneumonia; HPIV, human parainfluenza virus; ICU, intensive care unit; IQR, interquartile range; NA, not applicable. †p values were calculated by comparing total numbers of severe HPIV-associated pneumonia and severe influenza virus-associated pneumonia. ‡Defined as the period from hospital admission to discharge from the hospital or death.

**Table 4:** Univariate and multivariate analyses of risk factors for severe human parainfluenza virus community- and healthcare-acquired pneumonia in adults at a tertiary hospital in Seoul, South Korea, 2010–2019*

Variable	Univariate analysis			Multivariate analysis	
	Unadjusted odds ratio (95% CI)	p value		Adjusted odds ratio (95% CI)	p value
Hospital-acquired HPIV	3.38 (1.67–6.84)	0.001		2.21 (1.00–4.86)	0.049
SOFA score	1.29 (1.16–1.43)	<0.001		1.29 (1.15–1.44)	<0.001
Hematologic malignancy	3.46 (1.63–7.33)	0.001		1.35 (0.53–3.40)	0.53
Fungal co-infection	4.38 (1.20–15.99)	0.03		4.64 (1.05–20.63)	0.04

*Analyses of risk factors associated with 90-day mortality rates for patients with severe HPIV pneumonia. HPIV, human parainfluenza virus; SOFA, sequential organ failure assessment.

## Discussion

We investigated the epidemiologic and clinical characteristics of severe HPIV-associated pneumonia in adults. HPIV was the leading cause of severe virus-associated HAP and the third most common cause of severe virus-associated CAP. Most of the study population comprised elderly patients with underlying illnesses, and ≈50% of patients had co-infections at the time of ICU admission. The 90-day mortality rate for patients with severe HPIV-associated pneumonia was comparable to that of the patients with severe influenza virus-associated pneumonia. Fungal co-infection was strongly correlated with increased death.

HPIV was identified as the leading viral pathogen responsible for severe virus-associated HAP, despite ranking third after influenza and rhinovirus as the most prevalent causes of virus-associated CAP. Those findings might signify a higher risk for HPIV exposure and nosocomial transmission in the hospital environment, especially when compared with influenza and rhinovirus. HPIV generally induces asymptomatic or mild upper respiratory infections in healthy adults (*3*,*14*,*15*), which could result in a delayed or missed diagnosis of HPIV infection in healthcare workers, patient guardians, and patients. Consequently, those asymptomatic carriers might transmit the virus to other patients in the hospital (*16*). Furthermore, HPIV has been shown to survive on experimentally contaminated nonporous surfaces for <10 hours (*17*,*18*), suggesting fomites and contaminated environments could act as vehicles for HPIV transmission (*18*–*20*). We have previously reported on environmental contamination during an outbreak of HPIV-3 in a hematology ward; ≈80% of swab samples from an index patient’s environment were positive for HPIV-3 RNA, despite the environment being swabbed 5 days after a negative PCR conversion of patient respiratory samples (*16*). This finding suggests that HPIV-3 could potentially spread in hospitals through fomite transmission. In contrast to influenza virus, no effective therapeutic agents or vaccines exist for HPIV.

We found that >50% of patients with severe HPIV-associated pneumonia had co-infections with pathogenic organisms; bacterial co-infections were more common than viral and fungal co-infections. The high rate of co-infection could be linked to the inclusion of a substantial number of HAP cases in the study. Influenza virus–associated pneumonia is often complicated by subsequent pneumonia from *S*. *aureus* or *S*. *pneumoniae* (*21*). In contrast, patients with severe HPIV-associated pneumonia are frequently co-infected with gram-negative bacilli, such as *P. aeruginosa* or *K. pneumoniae*. Fungi, especially *Aspergillus* spp., were also major co-pathogens and were significantly associated with a high 90-day mortality rate. Increasing evidence indicates that patients with severe influenza-associated pneumonia (7%–19%) (*22*,*23*) and COVID-19 (5%–30%) are at risk for invasive pulmonary aspergillosis (*24*). However, data on patients with HPIV-associated pneumonia remains scarce. Only a few studies have shown that invasive pulmonary aspergillosis might also occur in patients with noninfluenza respiratory virus–associated pneumonia, such as respiratory syncytial virus or HPIV, particularly among immunocompromised hosts (*25*,*26*). Because most patients with *Aspergillus* spp. co-infections had immunocompromising conditions in this study, this co-infection can be attributed to host factors rather than an inherent characteristic of HPIV. If the clinical course of a patient with severe HPIV-associated pneumonia continues to deteriorate, clinicians should consider the possibility of co-infection with other pathogens.

Although influenza can be treated with effective antiviral agents, such as oseltamivir and peramivir, no licensed effective therapeutic agents are available to treat HPIV infections. In this study, ribavirin and IVIG did not reduce death, indicating the need for novel therapeutic agents and preventive vaccines for HPIV. A phase III clinical trial of DAS-181 as a novel therapeutic agent for HPIV is ongoing (*27*,*28*). Until novel therapeutic agents or vaccines are developed, it is crucial to maintain contact precautions and environmental cleaning against HPIV in hospital settings.

The strength of this study lies in its comprehensive overview of severe HPIV-associated pneumonia over a decade. We have provided insights into various epidemiologic and clinical characteristics of severe HPIV-associated pneumonia, including the identification of causative serotypes according to the site of pneumonia acquisition. Moreover, our study highlights the relatively frequent incidences of co-infections in patients with severe HPIV-associated pneumonia, particularly focusing on the effects of fungal co-infections on mortality rates.

The first limitation of our study is that it was conducted exclusively at a single tertiary care center and focused on a highly selected subset of HPIV patients. Nonpneumonic patients and those with mild to moderate pneumonia were not included, limiting the generalizability of our findings. Second, HAP cases could have been misclassified according to our definition if patients had upper respiratory infections with asymptomatic or mild symptoms that were not documented upon admission. Third, we did not perform BAL for all patients in this study; therefore, viral pathogens might have been present as bystanders causing asymptomatic, persistent, and prolonged shedding. In addition, HPIV detection might have occurred by chance, possibly stemming from ongoing nosocomial transmission in hospitalized patients who had HAP caused by other pathogens. Fourth, the small numbers of HPIV-1, -2, and -4 infected patients in the study precluded the analyses of differences in clinical characteristics and outcomes among those HPIV serotypes. Finally, we did not perform a molecular study of HPIV. Thus, we could not evaluate the effects of molecular characteristics on clinical features even within the same serotype.

In conclusion, HPIV is the leading viral pathogen of severe HAP in adult patients. In this study, we found that the mortality rate was comparable to that of severe influenza virus–associated pneumonia, and fungal co-infections significantly contributed to the high mortality rate. Clinicians should consider the high likelihood of co-infection with other pathogens in patients with HPIV-associated pneumonia, and contact precautions and environmental cleaning must be used to prevent HPIV transmission in hospital settings.
